# Analysis of the Gastro-Intestinal Tract of Marine Mammals: A Multidisciplinary Approach with a New Multi-Sieves Tool

**DOI:** 10.3390/ani11061824

**Published:** 2021-06-18

**Authors:** Giorgia Corazzola, Matteo Baini, Carla Grattarola, Cristina Panti, Federica Marcer, Fulvio Garibaldi, Enrica Berio, Cecilia Mancusi, Matteo Galli, Sandro Mazzariol, Maria Cristina Fossi, Cinzia Centelleghe, Cristina Casalone

**Affiliations:** 1 Department of Comparative Biomedicine and Food Science, University of Padua, 35020 Legnaro, Italy; giorgia.corazzola@gmail.com (G.C.); sandro.mazzariol@unipd.it (S.M.); 2 Department of Physical Earth and Environmental Sciences, University of Siena, 53100 Siena, Italy; matteo.baini@unisi.it (M.B.); panti4@unisi.it (C.P.); galli13@student.unisi.it (M.G.); fossi@unisi.it (M.C.F.); 3 Istituto Zooprofilattico Sperimentale del Piemonte, Liguria e Valle d’Aosta, 10154 Turin, Italy; Carla.Grattarola@izsto.it (C.G.); cristina.casalone@izsto.it (C.C.); 4 Department of Animal Medicine, Production and Health University of Padova, 35020 Legnaro, Italy; federica.marcer@unipd.it; 5 Department of Earth Environment and Life Sciences, University of Genoa, 16132 Genoa, Italy; fulvio.garibaldi@unige.it; 6 Istituto Zooprofilattico Sperimentale del Piemonte, Liguria e Valle d’Aosta, 18100 Imperia, Italy; enrica.berio@izsto.it; 7 ARPAT, Environmental Protection Agency of Tuscany Region, 57125 Livorno, Italy; cecilia.mancusi@arpat.toscana.it

**Keywords:** gastro-intestinal tract analysis, marine mammals, marine litter, micro-plastic, post-mortem investigations, diet analysis, parasitology

## Abstract

**Simple Summary:**

Currently procedures used to obtain samples from the gastro-intestinal tract (GIT) and protocols used to perform their respective analyses do not allow a multidisciplinary approach of this system. In fact, the investigations applied on the GIT, when performed singularly, could impair or limit the other analyses, because the currently procedures do not consider the needs of other disciplines. This means that the analyses to perform must be selected a priori, sacrificing the collection of other types of data and leads to the risk of losing important information, especially for wildlife species. To solve this conflict, we implement and standardize a new methodological approach to the GIT of marine mammals, which allow the collection of samples for different disciplines at the same time, performing the respective analyses, interpret and compare their results in a multidisciplinary way. The compatibility of multiple analyses allows the gaining of more information about the cause of death of stranded marine mammals and to enhance the knowledge of their biology and ecology.

**Abstract:**

Organs and content of the gastro-intestinal tract (GIT) of marine mammals are relevant for a variety of investigations and provide data to researchers from different fields. Currently used protocols applied to the GIT for specific analysis limit the possibility to execute other investigations and important information could be lost. To ensure a proper sample collection and a multidisciplinary investigation of the GIT of marine mammals, a new multi-sieves tool and a specific protocol have been developed. This new device and approach allowed the simultaneous sampling of the GIT and its content for the main investigations concerned. The samples collected during these preliminary trials were suitable to perform all the different research procedures considered in this work. The obtained results show that with a few and easy procedural adjustments, a multidisciplinary sampling and evaluation of the GIT of marine mammals is possible. This will reduce the risk of losing important data aimed at understanding the cause of death of the animal, but also biology and ecology of marine mammals, and other important data for their conservation and habitats management.

## 1. Introduction

Monitoring and intervention on stranded marine mammals are unique opportunities to gain information about the single animal, the at-sea population and the habitat and area it lives, feeds and reproduces [1,2,3,4,5,6,7,8,9,10,11,12]. As recommended by international agreements and European environmental policies, a systematic and standardized approach to investigate stranded marine mammals is needed with the aim of a shared data collection and interpretation, and common conservational strategies [6].

A complete post-mortem examination and ancillary investigations should be based on international standardized protocols, taking into consideration the wide range of disciplines interested in and the need to investigate and interpret the collected information [6]. In fact, the post-mortem examination, besides the sampling necessary to determine the cause of death, guarantees the collection of other important data for marine mammal conservation and habitats management (e.g., genetic, interaction with anthropic activities, toxicological, age determination etc.). The need for standardized protocols is even more relevant with regard to the analysis of gastro-intestinal tract (GIT), because this system provides several samples and data to researchers for many fields, in comparison to other apparatus [13,14]. Indeed, the tissues and the content of stomach and intestine are relevant for a variety of investigations as pathological evaluation, diet analysis, marine litter detection, parasitological, microbiological, virological and algal biotoxins detection. The lack of a multidisciplinary and standardized approach to the analysis of GIT became even more relevant after the increase of the global concern about the ingestion of marine litter by marine megafauna. The monitoring of this phenomenon arise the need to sample the content of the GIT for the assessment of marine litter presence and its consequences [13,15,16,17,18]. This may direct inform upon cause of death and stranding, but also to gain useful information to the implementation of EU Directives (i.e., the Marine Strategy Framework Directive (MSFD), 2008/56/EU) or the resolution of international agreements.

The investigations applied on the GIT, when performed singularly, could impair or limit the other analyses, because the currently procedures do not take into account the needs of other disciplines. This means that the analyses to perform must be selected a priori, sacrificing the collection of other types of data and risking the loss of important information about the cause of death and the health status of the animal, marine mammals’ biology and ecology, and other important data for their conservation and habitats management [19]. For instance: Pathologists should open the GIT to collect relevant information on pathological changes, parasites burden and content’s features as well as samples for ancillary investigations; without a standardized approach which considers the needs of marine litter exam, this analysis could be impaired facilitating GIT content contamination by environmental micro-litter items. Parasitological and diet analyses, as marine litter assessment need to process the entire content; the execution of one of them prevent therefore the possibility to use GIT content for the other two investigations. In fact, micro-litter analysis requires the destruction of the organic matter of the sample, avoiding subsequent research such as diet evaluation and parasitology. On the other side, protocols for diet and parasitological analyses could prevent the collection of all micro-litter items and contaminate with environmental particles the sample. Moreover, dividing the GIT content in three parts for the aforementioned analysis would not allow homogeneous and representative sub-samples.

Scientific literature reporting ingestion of macro- and meso-litter is growing in number, but its effects on the health of marine mammals and the predisposing factors of ingestion are still poorly understood [20,21,22,23]. A standardized interpretation of the presence of macro- and meso-litter item in the GIT of cetaceans was proposed by the best practice document on post-mortem investigations, edited by ACCOMBAMS and ASCOBANS [6]. Some gaps remain for micro-litter studies since the absence of a standardized methodology for sampling, storing and sample treatment [18,19,24,25].

Furthermore, GITs analyses of large or medium-sized species are often difficult to perform with the existing protocols and tools, due to the large volume of content that must be evaluated and sampled [26].

The aim of this study is to fill these procedural gaps by creating a specific tool for the GIT opening, study and sampling, composed of a series of different meshes. This new device allows the sampling for the execution of multiple investigations on the same GIT, which are in conflict when executed through the contemporary procedures. In association, we implement a dedicated protocol that permits a complete and multidisciplinary investigation of the GIT of stranded marine mammals, giving the opportunity to collect, relate and interpret data from different disciplines on the same animal. The use of this new tool allows the performance of several investigations at the same time, such as pathological evaluation, diet and parasitological analyses, marine litter and algal biotoxin detection, microbiological and virological investigations. This approach to data collection from the GIT could enhance the possibility to assess the cause of death of the examined individuals, the knowledge of marine mammal biology and ecology, and the understanding of which are the best policies to adopt for their conservation.

## 2. Materials and Methods

To ensure a multidisciplinary investigation and a proper sample collection of the GIT of marine mammals belonging to species of every size, a new multi-sieves tool and its protocol of use has been developed, according to the following needs:

- To ensure the best practicality of use of the device;
- To ensure a fast GIT evaluation and sample collection, even in the cases of large volume of GIT content;
- To ensure the collection of samples suitable for the analysis in the different disciplines concerned (i.e., pathological evaluation, microbiological and virological analyses, algal biotoxin detection, diet and parasitological investigations, and marine litter presence assessment).
  Pathological evaluation and virological analysis are performed on the organs, microbiological analysis can be performed both on the organ or content before the separation though the sieves, algal biotoxins detection is performed on part of the content before the separation, while diet and parasitological analyses, and marine litter assessment are performed on the content. The presence of parasites that developed in the organ walls (e.g., Pholeter gastrophilus) is assessed during the pathological evaluation.

To reach these goals, 4 testing sessions were performed at the necropsy room of the Department of Comparative Biomedicine and Food Science of the University of Padua, in the two-year period 2018–2020, by a team composed of veterinary pathologists and parasitologists, ecotoxicologist and biologists. During these sessions different sieves meshes sequences were tested and the protocol was defined, through the evaluation of GITs of 5 cetaceans of different species.

### 2.1. Animals and GITs Analyzed

9 organs (5 stomachs and 4 intestines) of the GITs of 5 cetaceans (1 *Ziphius cavirostris*, 1 *Globicephala melas*, 1 *Stenella coeruleoalba* and 2 *Tursiops truncatus*) and their content were analyzed. All the animals stranded along the Italian coastline and were rescued by the Italian Stranding Network. The biological and stranding data, the hypothesis of the cause of death and the organs examined are reported in Table 1. The animals were selected according to their availability, their decomposition condition code (DCC) and the species.

All the GITs were frozen waiting the testing sessions to analyze them.

### 2.2. The Multi-Sieves Tool

**Figure 1:**
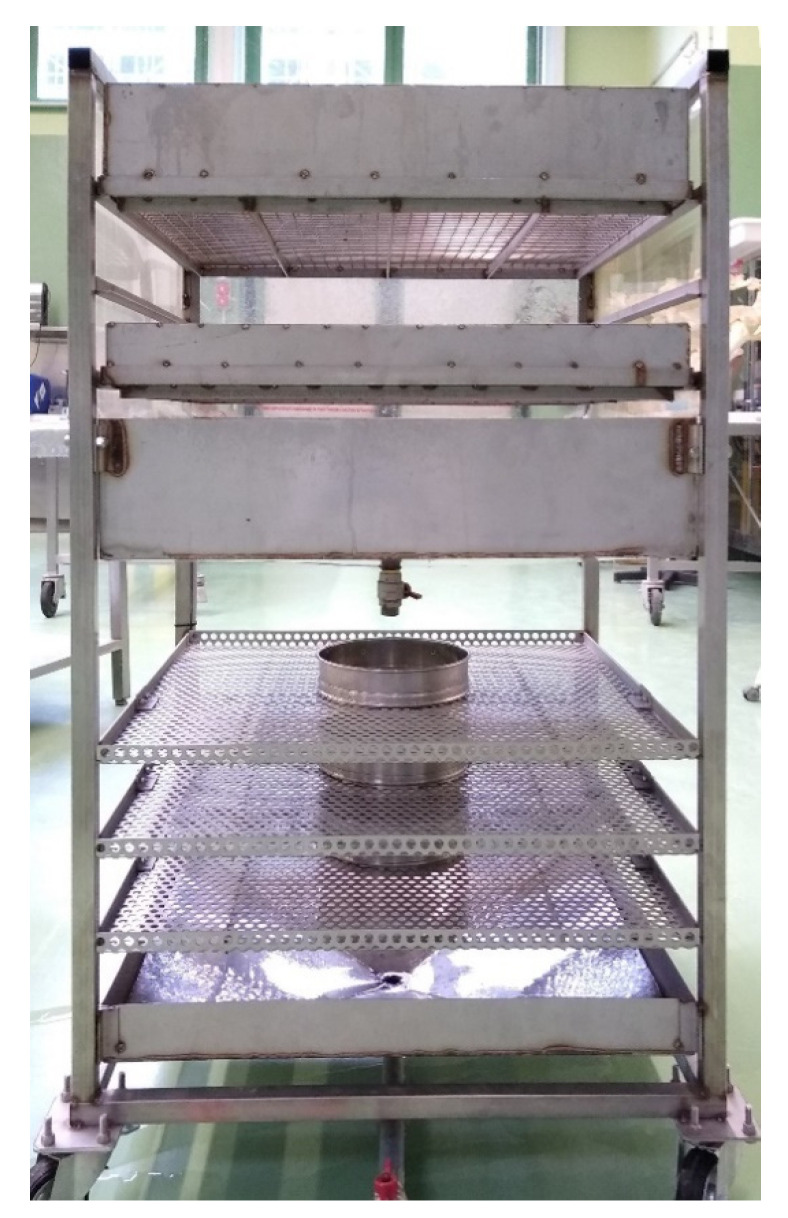
The multi-sieves tool is composed of the support and insertable sieves.

To identify the proper sieves sequence which allows the best practicality, a fast execution of the protocol and an optimal splitting of the GIT content for a correct visualization of its components, different meshes orders were inserted on a specific support, created ad hoc (Figure 1) and tested through the evaluation of the cetaceans GITs listed in Table 1.

The meshes dimensions selected to tested in the sieves sequence and the motivation for which they were chosen, are reported in Table 2, as the possible investigation that can be performed on the content collected in the sieves. In Table 3 are shown the meshes sequences tested and the problems and solutions identified for each sequence.

### 2.3. Protocol for a Multidisciplinary Samples Collection and Analysis of the GIT

A standardized protocol for a multidisciplinary sample collection was implemented and refined during the testing sessions, with the aim of achieving the best practicality of use of the device, a fast execution of the GIT evaluation and sampling suitable for the main investigations concerned in the GIT analysis, accordingly to the stage of decomposition of the carcass and the temperature conservation (Table 4). The protocol development arises, as for the meshes sequence, from an iterative process of problems and relative solutions found during the testing sessions. To rinse the organ and to help the content going through the sieves tap water coming from a water pipe was used.

The preliminary actions and the consecutive steps of the protocol are summarized below (for more details and the photographic sequence of the steps, see Supplementary Materials).

According to Table 4, in frozen GITs microbiological and histopathological exams are limited, while algal biotoxin analysis cannot be performed.

The sealing with a string or strap of cranial and caudal portions of the organs is necessary to minimize the contamination of GIT content from environmental sources of micro-litter items and to avoid the mixing of the content. Before each GIT analysis, the device must be washed accurately and the environmental micro-litter items eventually present must be sampled and analyzed, as control sample of the process (see Supplementary Materials for more details about the control sample).

- Thoroughly rinse the external part of the organ (i.e., stomachs or intestine);
- Weigh the organ still closed;
- Place each organ in a tank/container;
- Collect fecal sample from the rectum for copro-microscopic examination for the detection of parasitic elements (i.e., eggs, larvae, cysts and oocysts) [31];
- Sample the content and the surface of the mucosa for microbiological culture, with a swab under aseptic conditions if there is a suspect of a gastro-intestinal diseases and/or sample the tissue from any lesion suspected of microbiological origin observed during gross examination on the wall, after the removal of the content;
- Open the organ longitudinally, throughout the entire length, using scissors or scalpels;
- Collect gastric content sample for toxins detection, if marine algal biotoxins are suspected;
- Gently rinse the mucosa with current water in the tank to collect the content;
- Check and record any gross lesion and the presence of parasitic elements;
- Collect samples for histological investigations;
- Collect samples for virological analysis (molecular testing or isolation on cell culture);
- Rinse intensely the mucosa with current water to facilitate the complete exit of material from the organ and collect it in the tank;
- Weigh the organs without the content and subtract it from the weight of organ still closed to obtain the content wet weight;
- Transfer the organ contents from the tank/container into the first sieve (20 mm mesh) and rinse the material to make it proceed towards the next sieves;
- After an abundant rinse, extract the 20 mm and 5 mm sieves from the support and sample any marine litter item, parasite and alimentary residues in 3 different containers;
- Open partially the valve of the first collection tank positioned under the 5 mm sieve and made the flow continue through the round sieves;
- If marine debris, parasites and alimentary residues visible to naked eye are present in the 1000 µm, record its presence and amount and collect them;
- Collect the material present in the 1000 µm, 500 µm, 250 µm and 100 µm sieves in 4 different containers;
- These samples should be passed between and processed by all the laboratories interested in the GIT analysis, except for the 100 µm one which is exclusively for micro-litter assessment; the marine litter analysis must be the last one to be executed, due to its destructive process.

## 3. Results

### 3.1. The Multi-Sieves Tool

The final meshes sequence (Table 3) selected is: 20 mm–5 mm–1000 µm–500 µm (optional, to be used when the volume of gastro-intestinal content is abundant, and a better separation of the material is necessary)–250 µm–100 µm.

This sequence allows a good splitting of the gastro-intestinal content for an easy observation and collection of marine litter, diet and parasites, an adequate time of execution of the protocol and an adequate final number of samples to analyze.

Regarding the support where the sieves are inserted, it has an innovative characteristic which is the possibility to regulate the flow of GIT content between the sieves, reducing the possibility of clogging (see step 15 of the protocol). In addition, to reduce the risk of environmental micro-litter contamination, the support is covered by plexiglass walls.

For the dimensions of the final support and other components, and for other details of the multi-sieve tool see the Supplementary Materials (File S1).

### 3.2. Protocol for a Multidisciplinary Samples Collection and Analysis of the GIT

The protocol has proven to be easy, fast and practical to apply, even with large volumes of content to analyze.

As shown in Table 4, the freezing process limit the analysis that can be performed on the GIT. In any case, the protocol is still applicable for most of the investigations considered in this work.

### 3.3. Sample Suitability for the Investigations Concerned in the Multidisciplinary Analysis

The following exams were performed in all the examined organs: pathological evaluation, diet and parasitological analyses, macro-, meso- and micro-litter presence assessment.

The protocol provides samples suitable to perform all the investigations executed on the samples collected in this work (i.e., pathological evaluation, virological analysis, diet and parasitological investigations, and marine litter assessment).

Regarding pathological evaluation, the visual inspection of the content and the subsequent record of alterations is guaranteed before the separation through the sieves. Moreover, the protocol enhances the observation and sampling of eventual gross lesions of the gastro-intestinal tissues and avoids the damage to the mucosa for histological analysis. Despite this, in all cases, the tissues were auto-lytic due to the freezing of the organs and in most of the cases, no histological lesions were recorded.

Considering Table 4, virological analyses were conducted only in animals 12694 and 12878, due to their DCC. Despite no lesions were observed, virological screening was performed, and it was negative for both the animals.

About diet analysis, the protocol guaranteed the visualization of macroscopic and microscopic food remains and the identification of diet composition, filtering the stomach and intestinal contents up to 250 µm. In 4 cases, food remains were highly digested, being represented by cephalopod beaks, fish bones and otoliths. The largest amount of food was found in the stomach contents of the *Z. cavirostris* (large number of squid beaks) and in the young *T. truncatus*, where many fish bones and otoliths belonging to the poor cod *Trisopterus minutus* were identified. Nevertheless, also small items were collected, both from gastric and intestinal tracts; in particular, very small cephalopod beaks were found in the intestine of the *S. coeruleoalba* specimen. Only one *T. truncatus* specimen showed a GIT completely empty of food remains.

As for diet, parasitological analyses were carried out on content of sieves up to 250 µm. In two organs (stomachs of the young *T. truncatus* and *G. melas*) the suboptimal preservation conditions of the sample, probably together with the physical damage due to the strong water flow used to process the GIT content, prevented the identification of the parasite aside from a generic assignment of the Phyla. One animal was negative for any gastro-intestinal helminth species, confirmed by the absence of parasitic elements in the fecal sample. Examination of the gastric walls during pathological examination revealed the presence of the trematode *Pholeter gastrophilus* included within typical nodular lesions in three animals (*S. coeruleoalba*, *T. truncatus* and *G. melas*). The copro-microscopic exam carried out on the stool of the examined animals confirmed the data obtained by the GIT analyses. No protozoan parasites were observed in fecal samples.

Regarding marine debris, at a first general evaluation of the content following the opening of the 1° stomach, the presence of a black and a transparent sheet-like items of marine litter were recorded, respectively in animal 12694 and 12878. The separation of the content through various sieves enhanced the visibility and sampling of macro- and meso-litter items eventually present in the content. The samples obtained from the protocol were suitable also for the micro-litter analysis, which was performed on samples used first for parasitological investigations and diet analysis. In fact, with the meshes sequence selected, smaller micro-litter items (<250 µm) are collected in the last sieve which has been exclusively included to collect its smallest fraction regardless other analysis (100 µm). Larger micro-litter items are, instead, obtained from the content of three other sieves (250 µm, 500 µm and 1000 µm), which, due to the destructive method (e.g., chemical/enzymatic digestion) necessary for its assessment, were used first for parasitological investigations and diet analysis.

Moreover, the protocol includes the one adopted by the MSFD for the assessment of marine litter in the GITs of marine animals, thus permitting that the data collected complies this EU Directive.

Microbiological examinations were not performed, due to the absence of suspected gastro-intestinal infective lesions; moreover, the gold standard for this analysis is culture which cannot be performed on frozen samples. Neither toxicological investigation focused on marine algal biotoxins detection were also attempted, due to the freezing of the organs. Despite this, is reasonable to assume that the suitability of these samples was kept, because the application of the protocol does not affect the sampling procedures for these types of analyses.

The general results obtained from the multiple investigations executed are summarized in Table 5.

## 4. Discussion

The main goal of this study was the development of a specific multi-sieved tool and its protocol of use, aimed to solve the conflict between the investigations applied on the GIT, which could impair or limit each other when performed with the currently procedures.

The new device and the related analytical approach facilitate and standardize the procedures for multidisciplinary investigations and sampling of marine mammal GIT and its content. Using this tool and its protocol of use, all the analyses listed can be performed at the same time, according to the DCC and preservation, guaranteeing the possibility to collect all the foreseen samples, perform the scheduled analyses, interpret and compare their results. If performed singularly, each of the above reported analyses, useful for a complete health assessment, would impair or limit other investigations. The synergic interaction of applying different disciplines on the same organ and its content and/or sample them is clear in the examples of micro-litter effects on marine organisms. In fact, the systematic correlation of results obtained from micro-litter analysis, pathological, microbiological and virological investigations on the GIT could enhance the understanding of the role of these particles as carrier of pathogens, possibly altering the intestinal microbiota and leading to GIT or systemic pathologies [25,32,33]. Its potential immunosuppressing effect, carrying harmful chemicals and releasing toxic plastic components, could be investigated through the comparison with toxicological analysis on the GIT and the application of the three-fold approach created by Fossi et al. [15], which consider the assessment of the presence of plastic tracers in the tissues and biomarkers for damages at molecular, cellular and histopathological level.

Furthermore, this new methodology could solve any issue related to the analysis of high volumes of gastro-intestinal contents usually difficult to manage and analyze, as those of fin and sperm whales.

The protocol gives the possibility to collect suitable samples for the analyses considered in this work and solve the conflict between diet evaluation, parasitological analysis and marine litter presence assessment. In fact, currently used methods for micro-plastics detection take advantage of organic matter dissolution, which certainly misses the aim of a parasitic and diet analysis [19,24,25]. On the other side, protocols for parasites isolation and diet evaluation could hamper the collection of all debris particles present in the sample, and contaminations from used tools could not be excluded in the quantification of micro-litter. This study gives the opportunity to harmonize the different analyses, sampling and preservation procedures and protocols. In fact, the same GIT content sample can be used after sieving for diet, parasitology and micro-litter analyses, which usually limit or exclude each other, also allowing a gross evaluation of the GIT walls by the pathologists and the subsequent collection of tissues for other investigations according to the current protocols and depending on DCC and temperature conservation [6].

Despite the protocol guarantees the application of different investigations on the same sample, the work presents some limitations that needs to be considered. The freezing process was necessary to wait the testing sessions where analyze the GITs, but this has limited the histopathological evaluation, as well as the gold standard for microbiological investigations, and exclude the algal biotoxin detection. Despite this, it is reasonable to assume that the suitability of these samples was kept, because the application of the protocol does not affect the sampling procedures for these types of analyses. Furthermore, the small number of organs evaluated do not allow any significant correlation among the GIT analysis results, neither between them nor other results obtained from the post-mortem examination. A systematic use of this new tool and the future analysis of a higher number of GITs is required to allow this kind of consideration.

Considering results obtained from the three aforementioned investigations, parasitological analysis procedures used in this work do not differ too much from the ones routinely used. In fact, meshes of different sizes are used for the detection of gastro-intestinal helminths, according to host species and size of the expected parasites [30,34,35]. In the approach here applied, the helminth diversity observed corresponds to that commonly reported in the Mediterranean area for the diverse host species [36,37]. The collection of fecal samples from rectum is an important issue as it permits the obtaining of information regarding protozoan infection as well as confirming the presence of adult helminths. To maximize the results obtained for parasitological purposes we suggest being careful in the isolation of the pancreatic tissue before examination of GIT, in order not to include in this analysis parasites that usually are found in the glandular ducts. Moreover, during the flushing, water flow strength should be controlled so as not to damage the parasites, especially when preservation conditions of the parasites are expected to be suboptimal.

Regarding diet analysis, the results herein obtained provided the evidence that using a mesh of 250 µm it is possible to collect even the smallest highly digested hard parts of fish, cephalopods and other organisms present in the GIT, useful for the identification of prey. As well as for parasitological analysis; also, for the study of the diet a very important factor is the regulation of the water flow. A strong water flow, especially if applied on items on large mesh size sieves (i.e., 2 cm), could damage the soft tissues present and wash them out, thus altering the considerations on any recent meals, which could suggests a good health status and be helpful in interpreting post-mortem findings, in particular in by-caught animals [38]. It is suggested to gentle wash, remove and preserve any grossly detectable remains immediately after opening the stomach on the first sieve (2 cm mesh). Moreover, in studies on food and feeding habits of stranded cetaceans, the investigation on intestine contents is usually overlooked, mainly due to the great effort needed in comparison with the low probability to find food items. Considering that several cephalopod beaks have also been found in the intestine of one specimen out of the 5 analyzed in this study, the use of this device would facilitate the search for food items in this organ as possible source of information.

Regarding marine litter analysis, the obtained results turn out to be among the first coming from cetaceans of the Mediterranean Sea, which simultaneously analyze the presence of ingested macro-, meso- and micro-litter in all the different portions of the GIT. Micro-litter presence was shown in all the specimens considered representing the most abundant items of marine litter isolated, while only a small amount of macro- and meso-litter were reported exclusively in the stomach of *Z. cavirostris* and *S. coeruleoalba*. It should be noted that the simple general evaluation of the content allowed the visualization and sampling only for marine litter of greater size (i.e., the black and transparent sheet-like items). Control samples allowed the proper assessment of the background contamination during all stages of sample processing, from collection to the different analytical steps. The use of the device and the protocol adopted minimize the contamination of the samples as a few micro-litter (including micro-fibers) were detected in the control samples, making accurate the evaluation of ingested particles.

From these assumptions, arise that the device represents a new support to the diagnostic *iter* for the identification of the cause of death of cetaceans specimens, if associated with an exhaustive and complete post-mortem examination. Data acquired from the examination of the GIT should therefore be integrated to the results obtained from other organs and tissues, thus implementing the understanding of the life history of the animal and identifying any pathogenic agents or other pathogenic factors affecting the GIT, which could have contributed to the compromising of the health status of the animal. Furthermore, the results obtained from the GIT evaluation through this new device are useful to support and supplement all other kind of analysis falling outside the post-mortem examination.

The analysis of refrigerated GIT coming from fresh carcasses is necessary to implement this study, performing all the analysis considered in this work. Moreover, the analysis of a greater number of GITs is necessary for possible statistical correlations between the different disciplines.

Other aspects that should be considered is the impossibility to distinguish the origin of the micro-litter item in the presence of digested or partially digested preys and the possibility to discarded marine litter items with the GITs of recent-ingested preys. The first aspect is difficult to deepen, but an implementation of the protocol could be to analyze fresh preys to proper evaluate the contribution of secondary ingestion to the presence of marine litter.

## 5. Conclusions

This new methodology could lead to the discovery of new possible correlations between the disciplines interested in the GIT considered in this work. Furthermore, the new approach presented contributes to understanding more the effects and predisposing factors of the ingestion of marine litter by marine mammals, especially regarding micro-plastic. In fact, the systematic correlation of results obtained from the tool application could enhance the understanding of the role of these particles as carrier of pathogens, possibly altering the intestinal microbiota and leading to GIT or systemic pathologies [25,32,33].

In conclusion, the new multidisciplinary approach to the GIT presented in this work will reduce the risk of losing important information about the life history and the cause of death of the animal and will enhance the knowledge of biology and ecology of the at-sea population and the acquisition of other important data for their conservation and habitats management.

## Figures and Tables

**Table 1:** Biological and stranding information, hypothesis of the cause of death and organs tested. ID of animals refers to the Italian Stranding Database [27], DCC: decomposition condition code, which is the stage of decomposition of the carcass at the necropsy [6]; n.d.: not determined.

Animals ID	Species	Stranding Date and Location	Estimated Age	Cause of Death Hypothesis	DCC	Organs Tested	Conservation
12694	*Z. cavirostris*	22/12/17 Donoratico (LI)	Adult	Infectious (viral/parasitic)	2	Stomach	Frozen
12878	*S. coeruleoalba*	03/01/19 Alassio (SV)	Adult	Infectious (viral)	2	Stomach and intestine	Frozen
12755	*G. melas*	17/03/18 Aglientu (OR)	Adult	n.d.	3	Stomach and intestine	Frozen
12948	*T. truncatus*	14/04/19 Pellestrina (VE)	Young	n.d.	3	Stomach and intestine	Frozen
13065	*T. truncatus*	15/9/2019 Pellestrina (VE)	Adult	n.d.	3	Stomach and intestine	Frozen

**Table 2:** Mesh dimensions selection and justification for the choice they were selected. 20 mm, 10 mm, 5 mm and 2 mm sieves are composed of square meshes created ad hoc, 1000 μm, 500 μm, 250 μm and 100 μm sieves are round laboratory sieves. In the third column are reported the investigations which that can be performed on the content collected by each sieve.

Mesh Dimension	Justification for Choice	Investigations
**20 mm**	Retains undigested macro-food; separates the biggest marine debris items present.	Diet analysis, macro-litter assessment, parasitology
**10 mm**	Increases the content subdivision and improves its visualization.	
**5 mm**	Cut-off dimension between macro/meso- and micro-litter; increases the content subdivision and improves its visualization [28].	Diet analysis, meso-litter assessment, parasitology
**2 mm**	Increases the content subdivision and improves its visualization.	
**1000 μm**	Cut-off dimension for MSFD protocol for the analysis of micro-litter: in this way, the MSFD protocol is included in the new meshes device protocol [29].	Diet analysis, micro-litter assessment, parasitology
**500 μm**	The minimum size required for diet studies; increases the content subdivision and avoid clogging problems [6].	
**250 μm**	Size required for diet analysis which considers the presence of otoliths and beaks of small species and for collecting gastro-intestinal helminthes; increases the content subdivision and avoid clogging problems [6,30].	
**100 μm**	Collects only micro-litter items.	Micro-litter assessment
**53 μm**	Collects only micro-litter items.	

**Table 3:** Meshes size selection process. Mesh sequences tested during the practical sessions, problems encountered and respective solutions.

Mesh Sequence	Problems	Solutions
**20 mm–10 mm–5 mm–2 mm-1000 µm–500 µm–53 µm**	1. The time of execution of the protocol based on this number of sieves was too slow; 2. There was clogging problems at the 53 µm mesh level.	1. The number of square sieves decreased (the 10 mm mesh was eliminated); 2. The 500 µm mesh was substituted with a smaller one (250 µm) to have less material in the 53 µm mesh.
**20 mm–5 mm–2 mm–1000 μm–250 μm–53 μm**	1. The time of execution of the protocol based on this number of sieves was still too slow (the separation and visibility of the content were optimal); 2. The clogging problem was partially but not completely solved.	1. The 2 mm mesh was removed; 2. The 500 µm mesh was re-insert (in addition to the 250 µm) to have less material in the last sieve. The 53 µm mesh was substituted for a 100 µm one.
**20 mm–5 mm–1000 μm–500 μm–250 μm–100 μm**	1. With the re-insertion of the 500 µm mesh, the time of execution of the protocol remain too slow; 2. The problem of clogging was present only in large amounts of content, depending on the content composition.	1. The 500 µm became optional, to be used when the amount of gastro-intestinal content is abundant and a better separation of the material is necessary; 2. To totally solve this problem, the application of a shaking system or a manual shaking of the sieves for the smaller meshes could be necessary in some cases.
**20 mm–5 mm–1000 μm–500 μm–250 μm–100 μm**	**With this sequence, the main problems encountered were completely solved and it became the definitive mesh sequence.**	

**Table 4:** Analysis that can be performed on GIT organs according to the decomposition condition code (DCC) and conservation of both the organs and content. X = suitable, * = limited, ^Δ^ = limited to Polymerase Chain Reaction (PCR) analysis, microbiological cultures cannot be performed [6].

	DCC 1	DCC 2	DCC 3	DCC 4	DCC 5	Conservation (Refrigerated/Frozen)
Microbiological analysis (Organ and content)	**X**	**X**	*			Refrigerated/ frozen ^Δ^
Virological analysis (Organ)	**X**	**X**	*			Refrigerated/frozen
Anatomo-pathological macroscopic evaluation (Organ)	**X**	**X**	**X**	*		Refrigerated/frozen
Histopathological evaluation (Organ)	**X**	**X**	*			Refrigerated/frozen *
Parasitological analysis (Organ and content)	**X**	**X**	**X**	*		Refrigerated/frozen
Diet analysis (Content)	**X**	**X**	**X**	**X**	*	Refrigerated/frozen
Macro- and meso-litter presence assessment (Content)	**X**	**X**	**X**	**X**	*	Refrigerated/frozen
Micro-litter presence assessment (Content)	**X**	**X**	**X**	**X**		Refrigerated/frozen
Algal biotoxins analysis (Content)	**X**	**X**	*			Refrigerated

**Table 5:** General results of the analysis executed on the GITs of cetaceans. S: stomach, I: intestine.

ID/Organ	Pathological Evaluation	Diet Analysis	Parasitological Investigations	Marine Litter Assessment
12694/S	Gross findings: abundant content was present. Histological findings: severe diffuse autolysis of the tissue.	Presence of macro- and micro-food residues (squid beaks, eye lens and otoliths). More in details, 273 upper and 262 lower cephalopod beaks were present. Regarding the upper beaks, 1 belonged to the family Ommastrephidae, 262 belonged to the genus *Histioteuthis* and 2 belonged to *Octopoteuthis sicula*. Regarding the lower ones, the majority (257) belonged to the genus *Histioteuthis*. Remains of eye lens and shellfish were found.	Nematode larvae belonging to the Family Anisakidae: 7 specimens.	Macro-litter: 5 items; Meso-litter: 5 items; Micro-litter: 49 items.
12878/S	Gross finding: scarce content was present. 1° stomach: multifocal, mild, chronic ulcerative gastritis. 2° stomach showed multifocal, mild, chronic gastritis; several nodules caused by *Pholeter gastrophilus* were observed in main and pyloric chambers. Multifocal thickening of the mucosa at conjunction of the two stomachs were recorded. Histological findings: severe diffuse autolysis of the tissue.	Presence of macro- and micro-food residues (cephalopod beaks and eye lens) More in details, 8 upper and 11 lower cephalopod beaks were present, belonging to the family Ommastrephidae (*n* = 10) and one Loliginid squid. All food remains were completely digested, suggesting a non-recent meal. Some remains of the marine seagrass *Cymodocea nodosa* were also found, probably accidentally ingested in shallow waters before stranding.	Adult stage of digeneans (whole specimens and fragments) belonging to the Family Brachycladiidae: 38 specimens.	Macro-litter: 1 items; Meso-litter: 1 items; Micro-litter: 8 items.
12878/I	Gross findings: no macroscopic content was present. No lesions were recorded. Histological findings: severe diffuse autolysis of the tissue.	Several small cephalopod beaks (7 upper and 9 lower) were found. The analysis of lower beaks allowed identification of 6 ommastrephids squid 2 specimens of the pelagic sepiolidae *Heteroteuthis dipar*; one beak was not identified.	Adult stage of digeneans belonging to the Family Brachycladiidae (fragments): 4 specimens. Adult cestodes (whole specimens and scolexes) belonging to the Family Tetrabothriidae: 159 specimens.	Micro-litter: 4 items.
12755/S	Gross findings: scarce content was present. Nodules caused by *Pholeter gastrophilus* were recorded in glandular stomach. Histological findings: severe diffuse autolysis of the tissue.	Presence of macro-food residues (vertebrae and otoliths of bony fish and cephalopod beaks and eye lenses). Four vertebrae and one otolith were encountered. The four lower cephalopod beaks were represented by one large *Octopoteuthis sicula*, 1 *Histioteuthis reversa* and 1 small *H. bonnellii*. All food remains were completely digested, suggesting a non-recent meal.	Fragments of Nematoda parasites, not morphologically identified.	No items.
12755/I	Gross findings: no macroscopic content was present. No lesions were recorded. Histological findings: severe diffuse autolysis of the tissue.	Absence of food evidence.	Adult stage of digeneans belonging to the Family Brachycladiidae: 4 specimens. Adult cestodes belonging to the Family Tetrabothriidae: 2 specimens.	Micro-litter: 41 items.
12948/S	Gross findings: abundant content was present. 1° stomach: multifocal, mild, chronic ulcerative gastritis. Histological findings: severe diffuse autolysis of the tissue.	Among the macro-food residues, the presence of an undigested octopus (*Octopus vulgaris*) provided evidence of a recent meal. Other remains were highly digested; in de-tails, a large amount of fish bones and vertebrae were found, together with 63 otoliths, all belonging to the poor cod *Trisopterus minutus*, representing a total amount of about 38 specimens. Additionally, cephalopod beaks were present, 5 upper and 6 lower octopod beaks and one lower Ommastrephid beak.	Negative.	Micro-litter: 7 items.
12948/I	Gross findings: no macroscopic content was present. Histological findings: moderate diffuse autolysis, multifocal reactivity of the gastro-intestinal associated lymphoid tissue (GALT) of the submucosa.	Absence of food evidence.	Negative.	Micro-litter: 8 items.
13065/S	Gross findings: no macroscopic content was present. 2° stomach: multifocal, mild, chronic catarrhal gastritis (attributable to parasites). Histological findings: moderate diffuse autolysis, in the submucosa of 2° stomach multifocal aggregates of parasitic elements (larvae and eggs) are recorded, attributable to *Pholeter gastrophilus.*	Absence of food evidence.	Fragments of Nematoda parasites, not morphologically identified.	Micro-litter: 8 items.
13065/I	Gross findings: no macroscopic content was present. No lesions were recorded. Histological findings: severe diffuse autolysis of the tissue.	Absence of food evidence.	Negative.	Micro-litter: 36 items.

## Supplementary Materials

The following are available online at https://www.mdpi.com/article/10.3390/ani11061824/s1, File S1: Technical details of the multi-sieves tool. Extended version of the protocol with details of all steps and explanatory figures.
