# Supplementary material for: Analysis of the Gastro-Intestinal Tract of Marine Mammals: A Multidisciplinary Approach with a New Multi-Sieves Tool
Source: Animals (Basel). 2021 Jun 18;11(6):1824. doi: 10.3390/ani11061824 (PMC8234426; doi:10.3390/ani11061824)
Supplement: Supplementary file 1 [file animals-11-01824-s001.zip › animals-1234938-supplementary.pdf]

Supplementary

The protocol is reported below in more detail respect the main text. Some Figures have been inserted to facilitate the understanding of the various steps.

1. Before each multidisciplinary assessment of the GIT, wash accurately the meshes system device: rinse the collecting tanks and the entire support, rinse the sieves from the bottom to the top (Fig. S1);

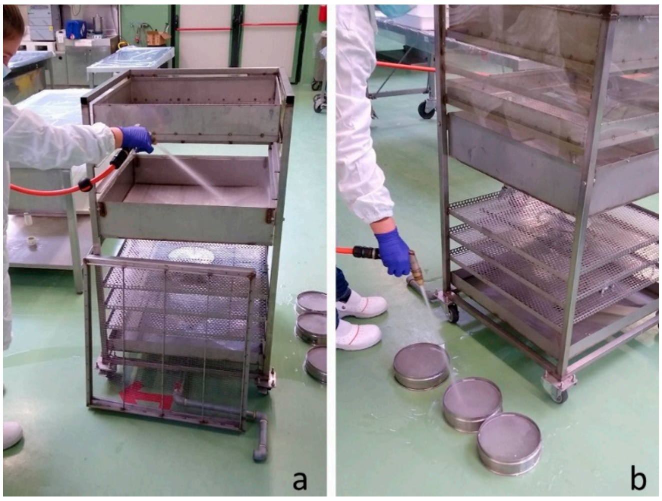

Figure S1 a) Rinse of the collecting tank; b) Rinse of the sieves, from the bottom to the top.

2. Control sample of the protocol: sample the environmental micro-litter items, which could contaminate the samples and invalidate the results (Fig. S2):
    - Run a little tap water into the support and collect it; run a little tap water in the 500 μm (if present), 250 μm and 100 μm sieves for the micro-litter items eventually present in the device;
    - Sample all the material which will be in contact with the samples (e.g. gloves, containers, clothes, strings/straps or other plastic objects)
    - During the session maintain a moistened cotton disc near the working area, which will collect the micro-litter items present in the air;

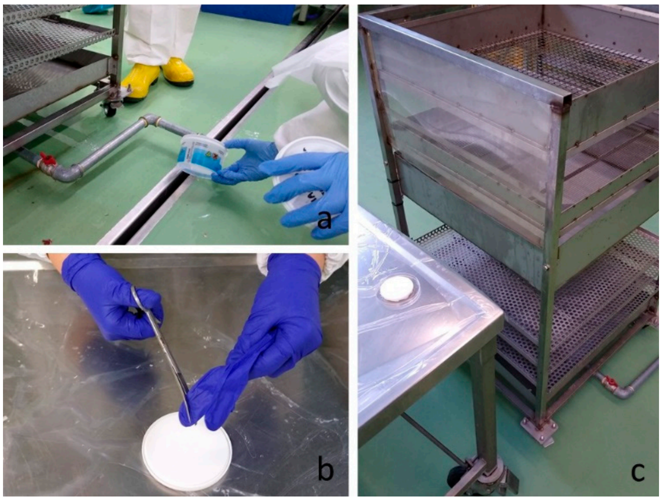

Figure S2 a) Collection of tap water passed through the support; b) Sampling of the material; c) Moistened cotton disk near the working area.

3. Prepare the organs: seal the cranial and caudal portion of the stomach and intestine with a string or strap (Fig. S3), separate the organs and thoroughly rinse the external part of the organs (Fig. S4);

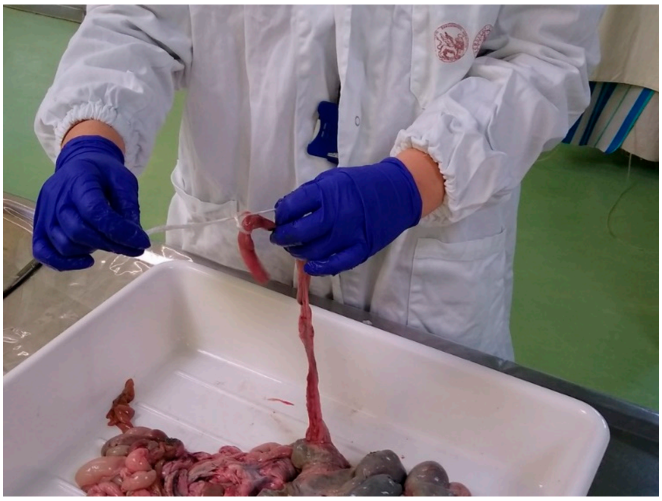

Figure S3 Sealing of the cranial and caudal portions of an intestine.

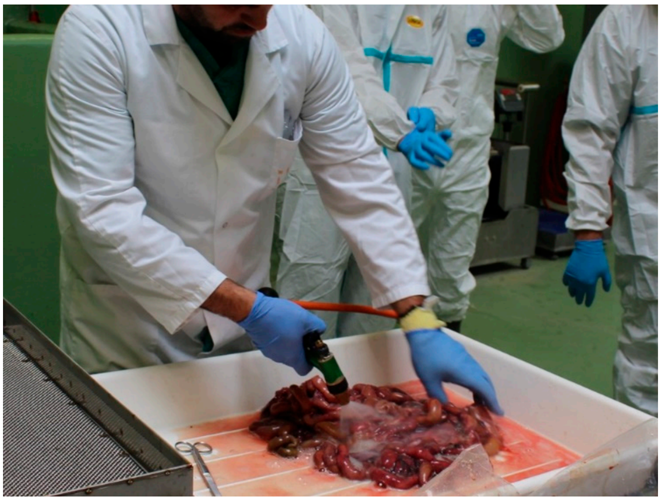

Figure S4 Rinse of the external part of an intestine.

4. Weigh the organs still closed;
5. Place each organ in a tank/container;
6. Collect faecal sample from rectum for copro-microscopic examination for the detection of parasitic elements (i.e. eggs, larvae, cysts and oocysts) (Marchiori et al., 2017) (Fig. S5);

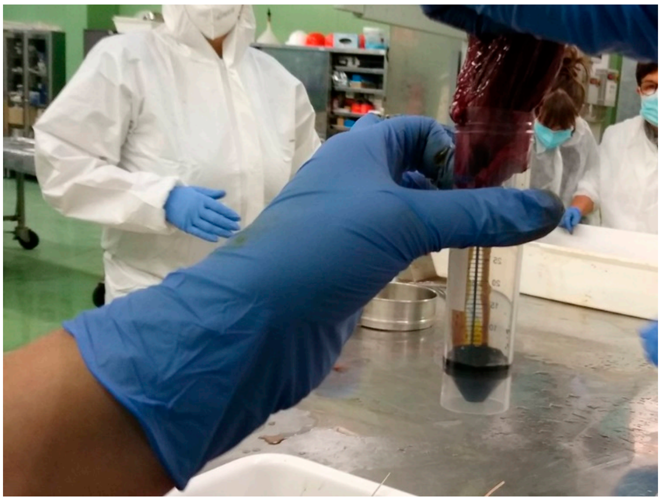

Figure S 5 Collection of the faecal sample.

7. Sample the organs for microbiological colture with a swab, under aseptic conditions if there is a suspect of a gastro-intestinal pathology; this sample can be collected even after opening the organ, in case of suspected gastro-intestinal bacterial lesion on the organ wall, as aseptically as possible (Fig. S6);

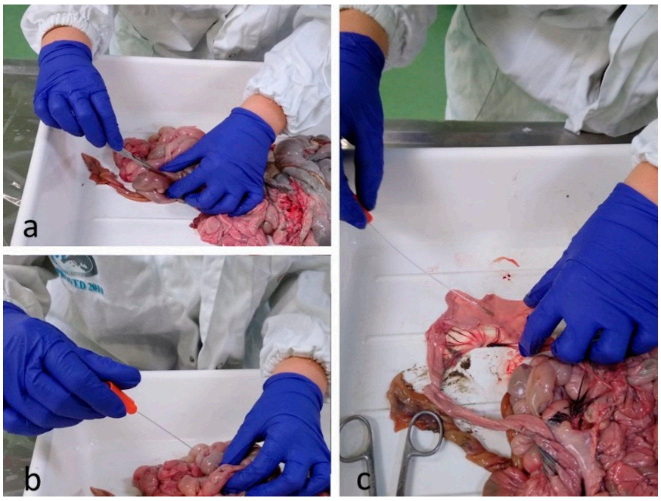

Figure S6 a) Opening of an intestine in aseptic conditions, after appropriate disinfection of the external wall; b) Sampling with a swab before the organ is fully opened; c) Sampling after opening the organ in case of lesions of suspected bacterial origin.

8. Open each organ longitudinally, throughout the entire length, using scissors or scalpels (Fig. S7);

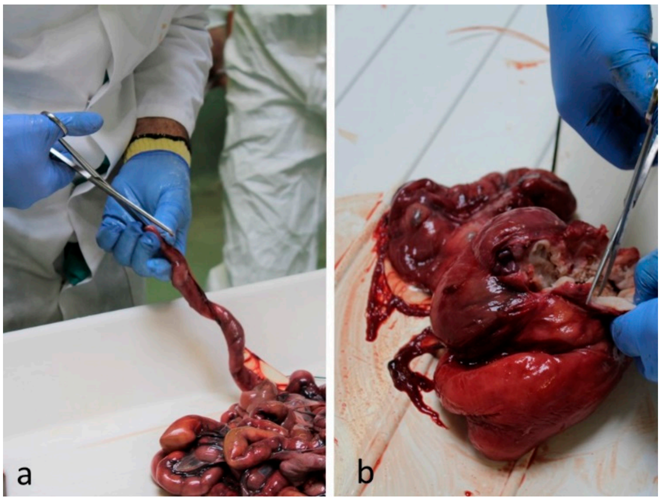

Figure S7 Opening the organ a) Opening of an intestine; b) Opening of a stomach.

9. Collect about 10 cc of gastric content, if a toxicological sample is needed (i. e. marine algal biotoxins detection);
10. Gently rinse the mucosa (Fig. S8);

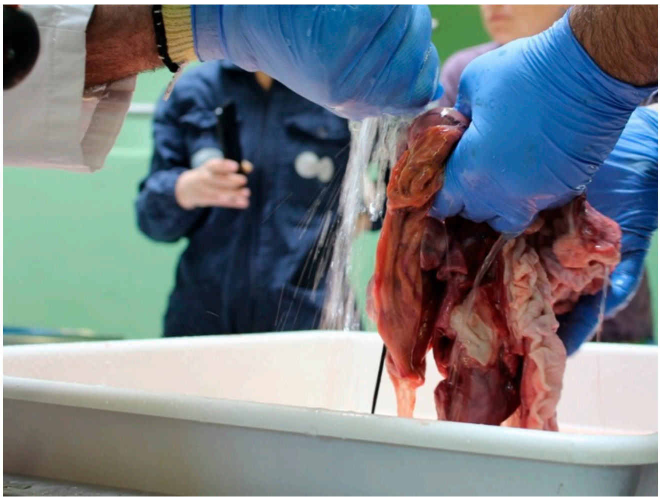

Figure S8 Gently rinse of the mucosa.

11. Check and record any gross lesion, evaluating at the same time the presence of parasitic elements (i.e. nodules caused by *Pholeter gastrophilus* and specimens of *Braunina cordiformis* embedded in stomach chambers (Fig. S9);

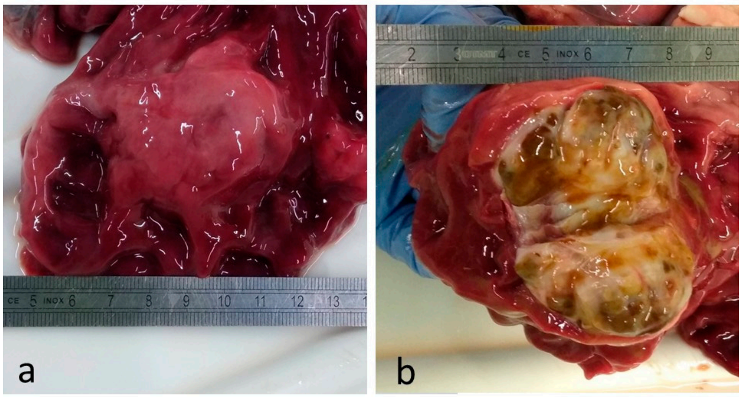

Figure S9 Photographic record of a lesion in the 2nd stomach of a specimen of *Stenella coreuleoalba*, attributable to *Pholeter gastrophilus*; a) focal lesion of 4 cm in diameter with a nodular appearance; b) cut surface of the lesion.

12. Collect samples for histopathological evaluation (microscopic examination, IHC investigations) and virological analysis (molecular testing and/or isolation on cell culture) (Fig. S10);

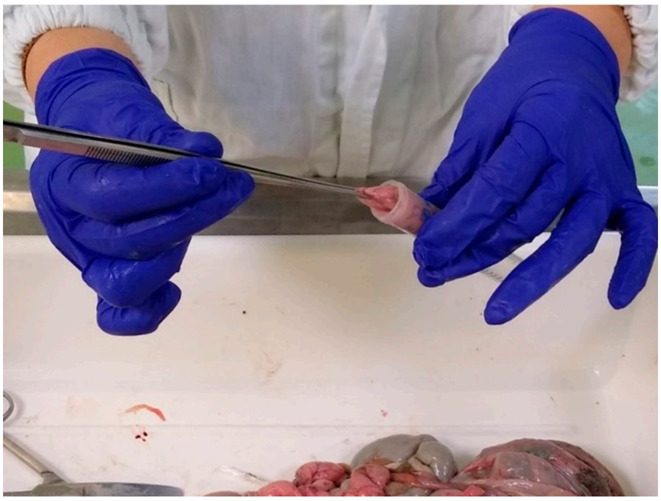

Figure S10 Sampling for virological investigations.

13. Rinse intensely the mucosa to facilitate the complete exit of material from the organ;
14. Weight the organs without the content and subtract it from the weight of organ still closed to get the content wet weight;
15. Transfer the organ contents from the tank/container into the first sieve (20 mm mesh) and rinse the material to make it proceed towards the next sieves (Fig. S11);

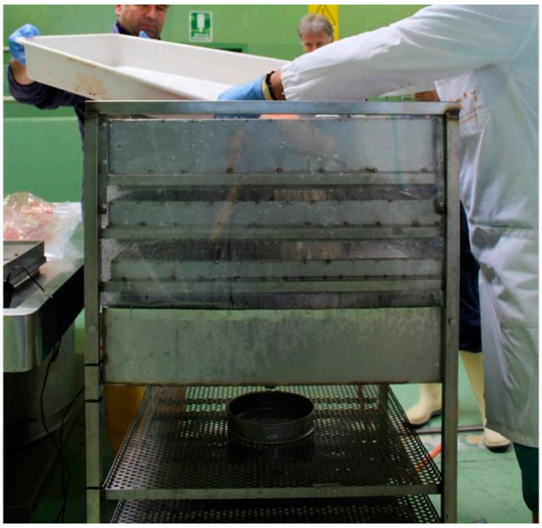

Figure S11 Transfer of the contents from the tank to the first sieve (mesh 20 mm).

16. After an abundant rinse, extract the 20 mm and 5 mm sieves form the support and sample any marine litter (Fig. S12), parasite and alimentary residues (Fig. S13) in 3 different containers;

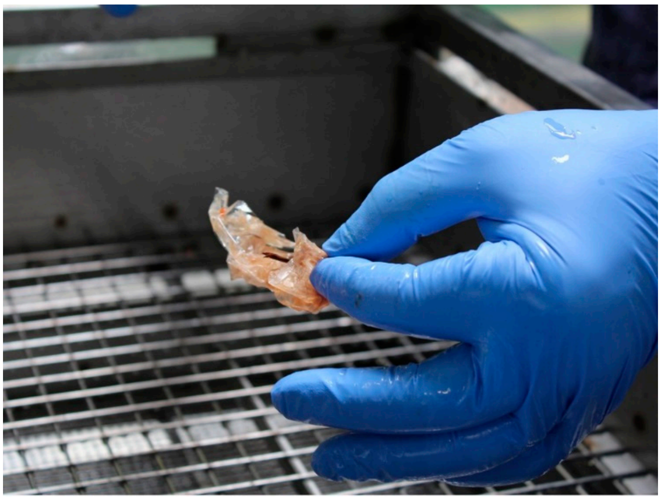

Figure S12 Collection of macro- and meso-litter from the first sieve (20 mm).

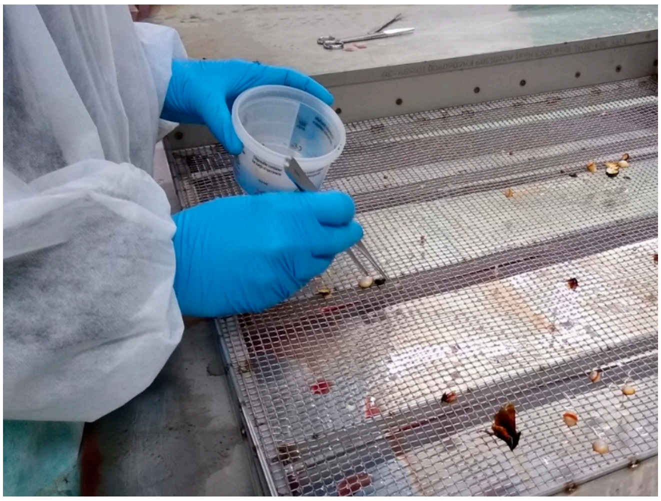

Figure S13 Collection of food macro-elements from the second sieve (50 mm mesh)

17. Open partially the valve of the first collection tank positioned under the 50 mm sieve and made the flow continue through the round sieves (Fig S 14); in case of clogging of one of the sieves, shake it gently without remove it from the support; alternatively place a hose directly on the bottom of the sieve and letting the water flow (this operation must be done gently, without scratching the mesh);

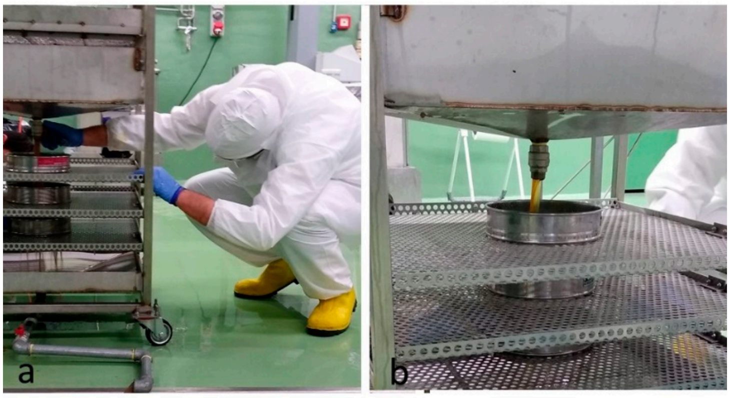

Figure S14 Opening of the valve located under the first collection tank to make the contents continue towards the circular sieves.

18. If marine litter, parasites and alimentary residues visible to naked eye are present, record the presence and amount, collect and add them to the containers of step 16 (Fig. S15);

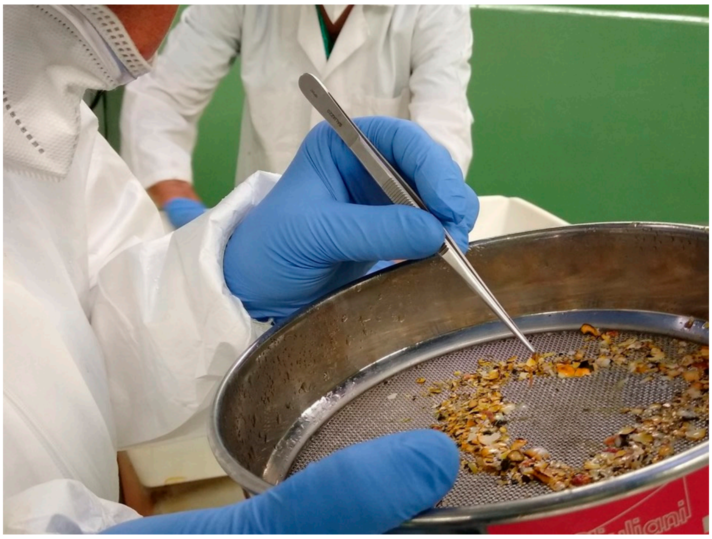

Figure S15 Removal of parasites visible to the naked eye from the third sieve (1000 µm mesh).

19. After the collection of items visible at naked eye, collect the residual material present in the 1000 µm, 500 µm, 250 µm and 100 µm sieves in 4 different containers (Fig. S16): insert the sieve upside down into a funnel and spray 70% alcohol solution on the surface of the sieve using a garden sprayer to collect all the contents in the container (Fig. S17);

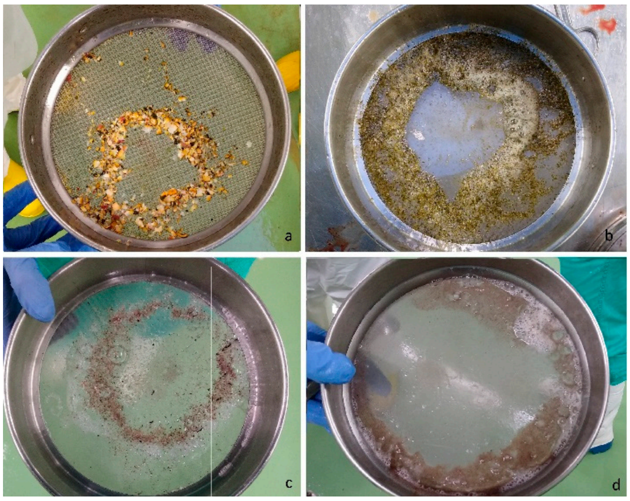

Figure S16 Content collected by circular sieves; a) 1000 µm; b) 500 µm; c) 250 µm; d) 100 µm.

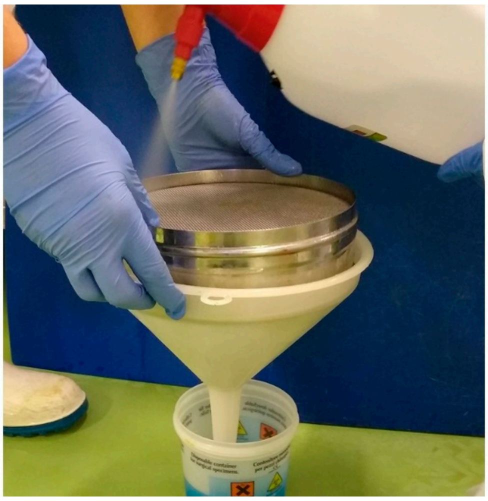

Figure S17 Collection of the content through the use of a garden sprayer

20. These 4 samples should be passed between and process by the laboratories for diet, parasitological and marine litter investigations; the marine litter items analysis have to be the last one to be execute, due to his destructive process. Laboratories for diet analysis and parasitological investigations have to process the sample with the precaution of minimising the risk of contamination by environmental micro-litter items.
